# Predicting Prostatic Obstruction and Bladder Outlet Dysfunction in Men with Lower Urinary Tract Symptoms and Small-to-Moderate Prostate Volume Using Noninvasive Diagnostic Tools

**DOI:** 10.3390/biomedicines13122894

**Published:** 2025-11-27

**Authors:** Jing-Hui Tian, Tsung-Cheng Hsieh, Hann-Chorng Kuo

**Affiliations:** 1 Department of Urology, Hualien Tzu Chi Hospital, Buddhist Tzu Chi Medical Foundation, Tzu Chi University, Hualien 970473, Taiwan; jhtian999@gmail.com; 2 Institute of Medical Sciences, Tzu Chi University, Hualien 970374, Taiwan; tchsieh@gms.tcu.edu.tw; 3 College of Medicine, Tzu Chi University, Hualien 970374, Taiwan

**Keywords:** bladder outlet obstruction, prostate, symptoms score, lower urinary tract dysfunction

## Abstract

**Objective:** The current study aimed to develop predictive models based on noninvasive clinical parameters to facilitate the early identification and stratification of patients with suspected bladder outlet dysfunction (BOD), thereby reducing the need for invasive diagnostic procedures. **Materials and Methods:** This retrospective study included 307 male patients with lower urinary tract symptoms (LUTS) refractory to medical therapy who were enrolled between January 2001 and May 2022. To assess the predictive performance of the model in an independent cohort, the dataset was randomly divided into the training set (70%) for model development and the test set (30%) for external validation. A two-stage modeling approach was adopted: Stage 1 involved detecting BOD, and stage 2 focused on identifying specific BOD subtypes. Backward stepwise logistic regression was conducted for model derivation, with internal validation performed using 5-fold cross-validation repeated 20 times. Clinical nomograms and a clinical decision-making framework were constructed based on the final modeling results. **Results:** In stage 1, the derived BOD model for detecting suspected BOD incorporated maximum flow rate, voided volume, intravesical prostatic protrusion (IPP), and prostatic urethral angle (PUA) as predictors. In stage 2, the derived benign prostatic obstruction (BPO) model included post-void residual (PVR), total prostate volume (TPV), and IPP as predictors. We also constructed nomogram to broadly screening BOD by the combination of maximum flow rate, voided volume, IPP, and PUA, a total score of ≥107 yielded the probability of 0.78 to identify BOD of 0.78. Subsequently, by combining PVR, TPV, and IPP, a total score of ≥39 yielded the probability of 0.35 to discriminate BPO. However, the BOD model (0.47) had a relatively low specificity, and the BPO model (0.58) had a lower sensitivity. Thus, these findings should be considered when applying the models in clinical practice. **Conclusions:** The results of this study revealed that using the clinical non-invasive parameters to create models can only yield a low sensitivity and low specificity for identifying BPO and the other BOD subtype. In patients with LUTS and small to moderate prostate volume, invasive video urodynamic study is still necessary when invasive treatment modality is recommended.

## 1. Introduction

Lower urinary tract symptoms (LUTS) are highly prevalent in men older than the middle age [1]. Clinical benign prostatic hyperplasia (BPH) is an umbrella term used to describe male LUTS. However, benign prostatic obstruction (BPO) accounts for the underlying pathophysiology in approximately 30% of men with LUTS [2]. In patients who did not respond to the initial alpha (α)-blocker therapy, the incidence of BPO in male LUTS is about 40% [3]. Bladder dysfunction and bladder outlet dysfunction (BOD) accounted for a high proportion of lower urinary tract dysfunctions (LUTD) other than BPO. These included detrusor overactivity (DO), detrusor underactivity (DU), hypersensitive bladder (HSB), bladder neck dysfunction (BND), dysfunctional voiding (DV), and poor relaxation of the external sphincter (PRES) [4].

In clinical practice, male LUTS is characterized by storage and voiding symptoms, which are frequently assessed using the International Prostatic Symptom Score (IPSS). The ratio of the voiding subscore to the storage subscore of IPSS can help classify men with LUTS as having either bladder dysfunction or BOD, guiding initial treatment with antimuscarinics or α-blocker, respectively [5]. Uroflowmetry, which is utilized to measure maximum flow rate (Qmax), voided volume, bladder capacity, and post-void residual (PVR), is the initial urological examination performed to classify BOD or non-BOD. Transrectal sonography of the prostate (TRSP), which is employed to measure total prostate volume (TPV), transition zone index (TZI), intravesical prostatic protrusion (IPP), and prostatic urethral angle (PUA), is commonly used to detect BPO and possible BND [6]. In patients suspected of urethral stricture or other pathologies, cystoscopy can also be used to detect bladder neck contracture, urethral stricture, bladder stone, or tumor. These office-based urological examinations have been widely used to obtain an initial diagnosis of BOD and perform follow-up on clinical response to medical treatment. However, their sensitivity and specificity are not always satisfactory. In cases where the desired treatment outcome is not achieved, urodynamic study (UDS) or video urodynamic study (VUDS) is recommended to accurately diagnose LUTD and provide appropriate treatment [2,3].

Although VUDS can precisely detect LUTD, its application in the initial assessment of male LUTS is limited by invasiveness and radiation hazard. Due to the current consensus on the diagnosis of male LUTS, UDS or VUDS has been considered as the later-line examination if the initial medical treatment has failed or invasive surgical procedure is planned [4,7]. Previous studies have attempted to establish a scoring system for predicting BPO using the parameters used in office-based urological examinations, based on the VUDS findings [6]. However, classifying BPO and non-BPO might not clearly define all LUTDs in men with LUTS. The subtypes of bladder dysfunction and BOD vary widely, and their treatment modalities differ. While previous studies tried to predict BPO or bladder outlet obstruction (BOO) by single parameter, a comprehensive, multi-stage model to identify BOD and differentiate BPO from other subtypes using non-invasive tools is lacking. It is mandatory important to search for a model to guide the accurate diagnosis of male LUTDs, without depending on invasive procedures such as UDS and VUDS.

Artificial intelligence (AI) refers to computational systems capable of executing tasks that traditionally require human cognition, including pattern recognition, prediction, and clinical decision support. Within healthcare, AI has been increasingly incorporated into diagnostic imaging, pathology, signal processing, and clinical risk stratification. Recent advances include deep-learning models for estimating functional connectivity from multichannel electroencephalography in schizophrenia [8], as well as machine-learning approaches that support hematologic and hematopathologic evaluations [9]. Collectively, these developments underscore AI’s expanding capacity to enhance diagnostic precision, automate complex analytical workflows, and inform clinical decision-making.

In urology, the application of AI and machine-learning methodologies to complex LUTDs has grown substantially. Prior studies have established CatBoost and XGBoost-based models to noninvasively differentiate BOO from detrusor underactivity among men with LUTS [10], and neural network models have been developed to diagnose female BOO using urodynamic study parameters [11]. AI-based approaches have also been employed to predict treatment failure and medication nonadherence in patients with overactive bladder [12]. These investigations collectively demonstrate the capacity of AI to improve disease subtype classification, diagnostic accuracy, and clinical workflow efficiency in urological practice.

Against this background, the present study applies AI-based analytical frameworks to develop noninvasive predictive models for BOD and its subtypes. Specifically, we seek to construct clinically interpretable prediction models and nomograms to distinguish BOD, particularly BPO, in men with LUTS, utilizing routinely obtainable clinical and urological assessment parameters.

## 2. Materials and Methods

### 2.1. Characteristics of the Patients

This retrospective study included 307 patients, and data were collected from January 2001 to May 2022. The inclusion criteria were male patients with LUTS who exhibited poor response after >3 months of continuous pharmacological treatment with agents including α-blockers, 5α-reductase inhibitors, and antimuscarinic agents. The exclusion criteria included patients aged <20 years, those with acute or chronic urinary retention, those with BPH with a high TPV (>60 mL), those with BOD caused by detrusor–sphincter dyssynergia, those with a history of lower urinary tract surgery, those previously diagnosed with urological malignancy, those with a subsequent pathological diagnosis of prostate cancer or bladder cancer, and those who did not undergo VUDS. The Research Ethics Committee of Hualien Tzu Chi Hospital, Buddhist Tzu Chi Medical Foundation, approved this study (approval number: 111-132-B). Considering the retrospective nature of the study, the need for informed consent was waived.

### 2.2. Noninvasive Assessment Variables and VUDS

All patients with LUTS underwent a standardized urological evaluation, including medical history review, physical examination, completion of the IPSS, prostate-specific antigen test, TRSP measurement, and uroflowmetry, before VUDS. In total, eight noninvasive clinical variables, including age, Qmax, voided volume, and PVR, were selected as potential factors for model development. The prostate-related parameters obtained using TRSP included TPV, TZI, IPP, and PUA.

**Figure 1:**
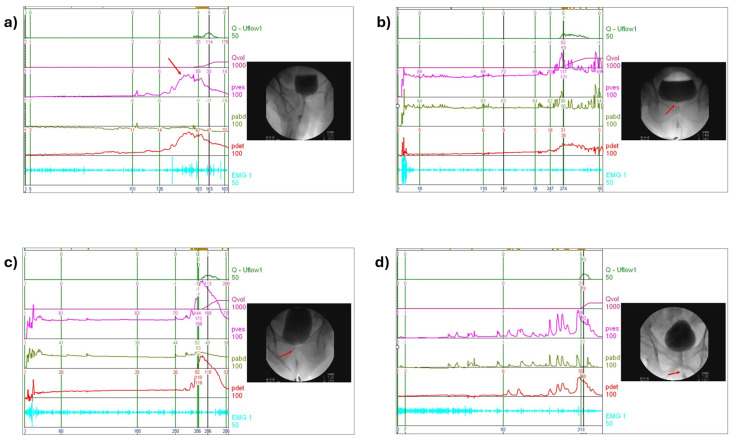
The videourodynamic tracings are representative of the following: (**a**) detrusor overactivity (DO) without bladder outlet dysfunction (BOD), (**b**) DO and bladder neck dysfunction (BND), (**c**) DO and benign prostatic obstruction (BPO), and (**d**) dysfunctional voiding (DV) without BPO.

All VUDS procedures were performed based on the standard operating protocols recommended by the International Continence Society and the Society of Urodynamics and Female Urology [13]. The VUDS parameters recorded during the filling phase included the first sensation of filling, full sensation, urgency sensation, and cystometric bladder capacity. The voiding phase parameters included the detrusor pressure during voiding, Qmax, voided volume, and PVR. Cinefluoroscopy with voiding cystourethrography was performed simultaneously, and the diagnosis of the LUTD subtype was based on a combined assessment of both imaging and urodynamic findings. Figure 1 shows the definition of the different subtypes of LUTD and the characteristic VUDS tracings.

### 2.3. Definition of the Prediction Targets

A two-stage modeling approach was developed to detect the five distinct subtypes of BOD, including BPO, BND, DV, and PRES, which are associated with different etiologies. All BOD diagnoses were determined based on the VUDS findings, which are a combination of imaging and urodynamic results, as described in a previous study [14]. In stage 1 (model 1), the model was designed to detect the presence of BOD, defined as the presence any of the five subtypes. In stage 2, for patients with BOD, separate models were constructed to classify each specific subtype: model 2 (BND) for BND versus non-BND, model 3 (BPO) for BPO versus non-BPO, model 4 (DV) for DV versus non-DV, and model 5 (PRES) for PRES versus non-PRES.

### 2.4. Synthetic Oversampling for Imbalanced Data

Due to the substantial class imbalance in the training data for the DV and PRES classification models, the minority classes included only 17 and 15 cases, respectively. This imbalance resulted in certain cross-validation folds with no positive cases, thereby compromising the model stability and performance assessment. To address this issue, the synthetic minority over-sampling technique (SMOTE) was applied to augment the minority classes. To prevent excessive oversampling and preserve a degree of real-world imbalance, the minority-to-majority class ratio was set to 1:4. This process generated 40 synthetic DV samples and 41 synthetic PRES samples. Model training and performance evaluation were then conducted using these SMOTE-adjusted datasets.

### 2.5. Model Derivation and Internal Validation

The cohort was randomly divided into the training set (70%) and the testing set (30%) for model development and validation, respectively. Logistic regression with backward stepwise selection, guided by the Akaike Information Criterion, was applied to the training set to derive the predictive model. Discriminative performance was quantified by the area under the receiver operating characteristic curve (AUC). Additional metrics—including accuracy, sensitivity, specificity, positive predictive value (PPV), and negative predictive value (NPV)—were calculated. The optimal probability threshold was determined using the Youden Index.

To enhance robustness and reduce bias, a 5-fold cross-validation was repeatedly performed 20 times within the training set, and the average performance across iterations was used to guide model selection. The final model was subsequently evaluated using the independent testing set to assess its generalizability.

### 2.6. Nomogram

In the nomogram, each variable’s score was determined according to the magnitude of its regression coefficient in the logistic regression model. Variables with larger coefficients were assigned a wider point range, while those with smaller coefficients were given a narrower range. Consequently, the contribution of each predictor to the total score varied, which was reflected by the different scale lengths and maximum point values in the nomogram. The logistic regression model derived from the training set was then used to construct a clinical nomogram for visualization based on the estimated β coefficients. In addition, the nomogram was internally validated and bias-corrected through 1000 bootstrap resampling procedures to assess its predictive accuracy.

### 2.7. Statistical Analysis

Categorical variables were presented as frequencies and percentages, and group differences were assessed using the chi-square test. Continuous variables were expressed as means with standard deviations. Between-group comparisons were conducted using the independent samples *t*-test. Comparisons among more than two groups were performed using one-way analysis of variance. Logistic regression models were used to predict binary outcomes, with model goodness-of-fit evaluated using the Hosmer–Lemeshow test. All statistical tests were two-sided, and a *p*-value of <0.05 was considered statistically significant. Data preprocessing and statistical analyses were performed with the Statistical Package for the Social Sciences software (version 27.0). Model development and nomogram construction were implemented using the *glmnet* and *rms* packages in R (version 4.1.1)

## 3. Results

### 3.1. Characteristics of the Participants

This analysis included 307 male patients with LUTS and suspected BOD who underwent VUDS. The dataset was randomly divided into the training set (70%, *n* = 215) and the test set (30%, *n* = 92). The overall mean age (±SD) of the participants was 67.8 ± 9.7 years. Of all the participants, 252 (82.1%) were diagnosed with a BOD subtype. Among these patients with BOD, only 87 (34.5%) had BPO. Meanwhile, the remaining patients (*n* = 165, 65.5%) were diagnosed with BND, DV, or PRES. There were no significant differences in the baseline demographic or clinical characteristics between the training and test sets (Table 1).

Based on a comparison of clinical characteristics, patients with the BPO subtypes were older, had a lower Qmax and voided volume, and had a higher TPV, TZI, IPP, and PUA compared with those with the non-BOD subtypes. Further, among the patients with the non-BOD subtypes, those with DU had a higher PVR and a lower TPV compared with those with the other subtypes. Patients with HSB were younger, had a higher Qmax, and had a lower PVR, TPV, TZI, and IPP than those with the other subtypes (Table S1).

Table 2 presents the baseline clinical parameters of the patients with different BOD subtypes and those with non-BOD subtypes. All baseline clinical parameters except PVR significantly differed among the five BOD subtypes (*p* < 0.05). Patients with BPO were older and had a lower Qmax, decreased voided volume, and greater prostate parameter value than those with other BOD subtypes and non-BOD subtypes.

### 3.2. Predictors of BOD and Each BOD Subtype

Table 3 presents the multivariable logistic regression models developed for the five different prediction targets. All models had good calibration, as indicated by the nonsignificant Hosmer–Lemeshow test results (*p* > 0.05). The BOD model was constructed in stage 1 to identify overall bladder outlet dysfunction from the general population. Four potential predictors were identified: Qmax (odds ratio [OR] = 0.925, *p* = 0.010), voided volume (OR = 1.004, *p* = 0.045), IPP (OR = 2.356, *p* = 0.117), and PUA (OR = 1.018, *p* = 0.118). Among these parameters, the Qmax and voided volume significantly differed. Subsequently, stage 2 models were developed exclusively for patients with BOD to further differentiate its subtypes. In the BND model, TPV (OR = 0.964, *p* = 0.006) and IPP (OR = 0.475, *p* = 0.082) were considered as potential predictors, with TPV reaching statistical significance. In the BPO model, the predictors included PVR (OR = 1.006, *p* = 0.081), TPV (OR = 1.064, *p* < 0.001), and IPP (OR = 2.254, *p* = 0.062), with TPV being statistically significant. The DV model, developed using SMOTE to address class imbalance, identified Qmax (OR = 1.105, *p* = 0.025), voided volume (OR = 0.990, *p* = 0.001), TPV (OR = 0.941, *p* = 0.006), TZI (OR = 20.051, *p* = 0.087), and PUA (OR = 0.958, *p* = 0.002) as predictors. Among these parameters, the Qmax, voided volume, TPV, and PUA significantly differed. For the PRES model, all selected predictors, including age (OR = 0.920, *p* < 0.001), voided volume (OR = 1.003, *p* = 0.041), PVR (OR = 0.988, *p* = 0.048), and TZI (OR = 0.032, *p* = 0.002), showed statistical significance.

Regarding the accuracy assessment and validation of the derived model (Table 4 and Figure S1), the stage 1 BOD model (model 1) achieved an AUC of 0.71 in the training set. Internal validation using 5-fold cross-validation yielded a comparable AUC (0.70 ± 0.10), indicating a stable performance. In the independent test set, the AUC and accuracy remained consistent at 0.70 and 0.74, respectively. The model maintained a high sensitivity (0.80) and PPV (0.87), despite having a specificity of 0.47 and an NPV of 0.35. The stage 2 model evaluation was subsequently conducted on patients with possible BOD. The BPO prediction model (model 3) achieved an AUC of 0.85 in the training set. Internal validation performed using 5-fold cross-validation yielded an AUC of 0.84 ± 0.07 and an accuracy of 0.81 ± 0.06, indicating good and stable discrimination between BPO and non-BPO cases. In the independent test set, the model achieved an AUC of 0.79, with an accuracy of 0.73, specificity of 0.82, sensitivity of 0.58, PPV of 0.63, and NPV of 0.78.

The PRES prediction model (model 5) achieved an AUC of 0.83. Internal validation showed an AUC of 0.81 ± 0.08, demonstrating that the model performance was consistent. In the independent test set, although the model maintained an acceptable overall performance, with an AUC of 0.73 and an accuracy of 0.80, both sensitivity (0.33) and PPV (0.15) dropped substantially, indicating instability in the model’s final predictions. Similarly, the BND and DV models (models 2 and 4) exhibited a substantially low AUC and accuracy in the independent test set, representing overfitting. As a result, only the BOD and BPO models were retained as the final tools for predicting BOD to support clinical decision-making.

### 3.3. Clinical Nomogram and Decision-Making Framework

**Figure 2:**
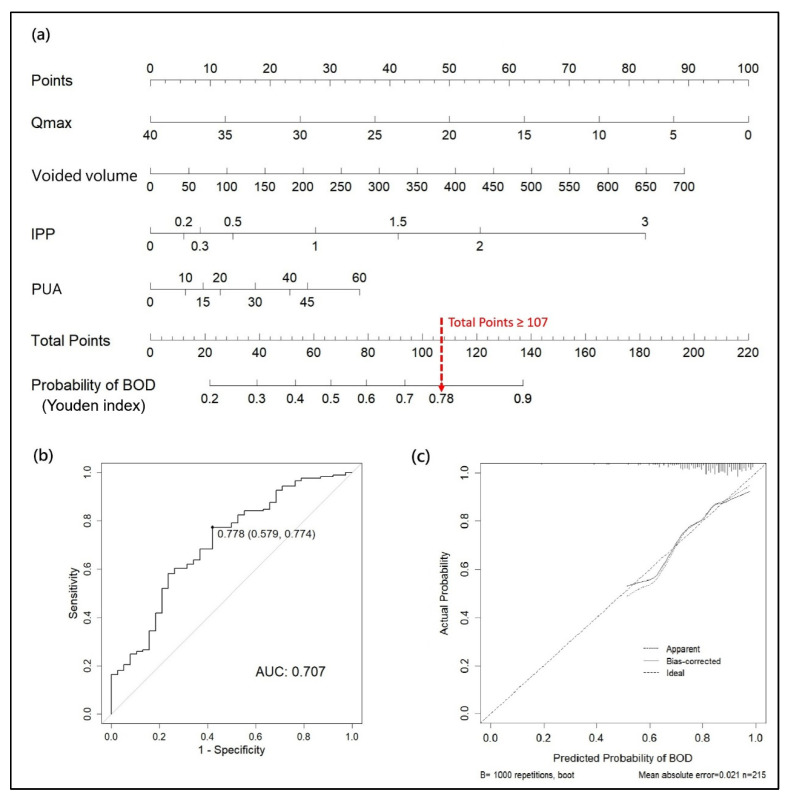
(**a**) Stage I: nomogram for predicting BOD. (**b**) ROC curve for the prediction of BOD using the clinical nomogram. (**c**) Calibration plot of the nomogram using 1000 bootstrap resamples. The solid line represents the apparent calibration. The dotted line indicates the ideal calibration. Qmax: maximum flow rate, IPP: intravesical prostatic protrusion, PUA: prostatic urethra angle, BOD: bladder outlet dysfunction.

Clinical nomograms were developed for predicting BOD and BPO based on the derived models of BOD (model 1) and BPO (model 3). For the stage 1 BOD model, a nomogram was developed based on four clinical variables: Qmax, voided volume, IPP, and PUA. A total score of ≥107 corresponded to a predicted probability of ≥0.78, representing the optimal threshold determined using the Youden index (Figure 2a). The nomogram had an AUC of 0.71 in the training dataset (Figure 2b). The calibration plot showed a good agreement between the predicted and observed probabilities, with a mean absolute error of only 0.021 (Figure 2c).

**Figure 3:**
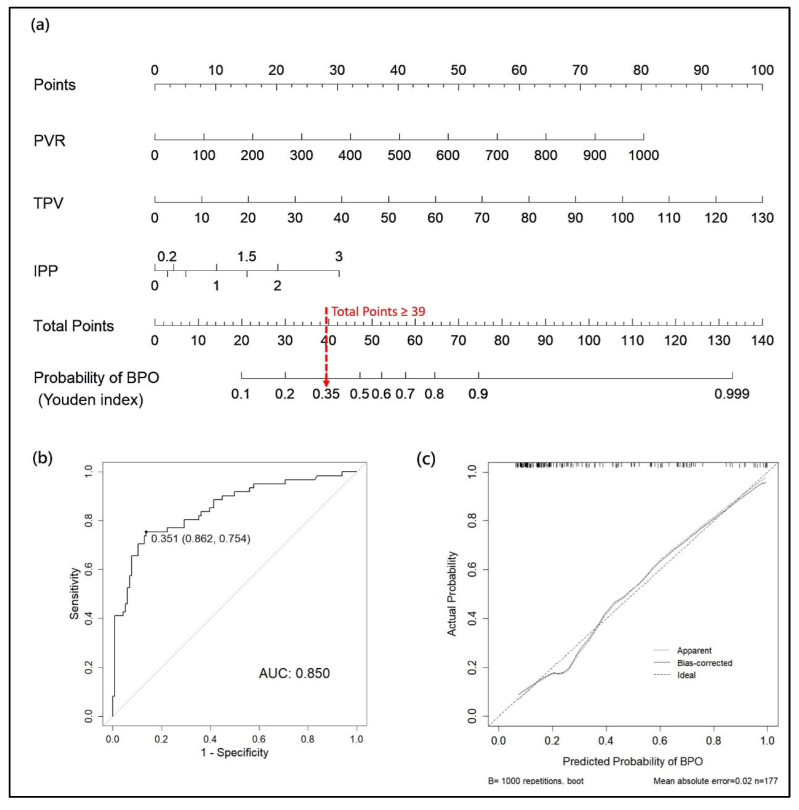
(**a**) Stage II: nomogram for predicting BPO in patients with BOD. (**b**) ROC curve for the prediction of BPO using the clinical nomogram. (**c**) Calibration plot of the nomogram using 1000 bootstrap resamples. The solid line represents the apparent calibration. The dotted line indicates the ideal calibration. PVR: post-void residual, TPV: total prostate volume, IPP: intravesical prostatic protrusion, BPO: benign prostate obstruction.

For the BPO model, a nomogram was developed based on three clinical variables: PVR, TPV, and IPP. A total score of ≥39 corresponded to a predicted probability of ≥0.35, which represents the optimal threshold determined using the Youden index (Figure 3a). The nomogram had an AUC of 0.85 in the training dataset (Figure 3b). The calibration plot showed an excellent agreement between the predicted and observed probabilities, with a mean absolute error of only 0.02 (Figure 3c).

**Figure 4:**
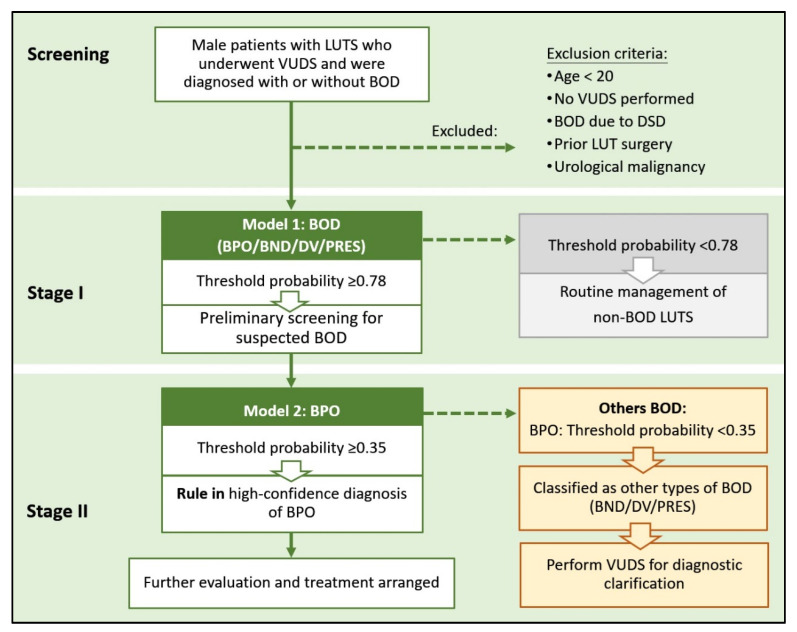
A two-stage model decision-making framework. Bladder outlet dysfunction (BOD) includes the following: benign prostate obstruction (BPO), bladder neck dysfunction (BND), dysfunctional voiding (DV), and poor external sphincter relaxation (PRES). VUDS: video urodynamic study.

The decision-making framework was further developed based on the BOD and BPO models to support clinical practice in diagnosing BOD and identifying specific BOD subtypes. As illustrated in the flowchart (Figure 4), the risk of BOD was first calculated using the clinical nomogram for BOD (Figure 2a) for any patient with suspected BOD. If this probability is equal to or exceeds the threshold of 0.78, the probability of BPO is then calculated using the clinical nomogram for BPO (Figure 3a). If this probability is equal to or exceeds the threshold of 0.35, the patient is considered to have BPO, and further evaluation and treatment are initiated as appropriate.

Based on the abovementioned results, although the diagnostic performance of each individual model may be insufficient to definitively confirm or rule out BOD, a stepwise stratification approach offers substantial clinical utility. In the initial stage, the BOD screening model, which is characterized by a high sensitivity, is used to broadly identify patients who are highly suspected of having BOD. In the second stage, the BPO model, which exhibits a high specificity, is applied to support diagnostic decision-making. Patients who are not classified as positive by either the BOD or BPO model are then directed to undergo further evaluation with VUDS. This dual-model strategy effectively reduces the need for invasive diagnostic procedures while maintaining diagnostic accuracy.

We also evaluated the sensitivity and specificity of single non-invasive clinical parameter for BPO in this study, including Qmax < 10 mL/s (56.3, 29.3), TPV ≥ 40 mL (66.7, 56.9), PUA ≥ 30 degrees (85.1, 41.1), IPP ≥ 0.5 cm (62.1, 61.4), and PVR ≥ 100 mL (17.2, 45.5). The sensitivity and specificity are not very satisfactory for a single parameter to identify BPO in men with LUTS and small to moderate prostate volume. Therefore, a nomogram for accurately diagnose BPO is warranted. The data are presented in Supplementary Table S2.

## 4. Discussion

The study results, for the first time, provide a reasonable two-stage model for predicting the presence of BOD and identifying BPO in male patients with LUTS and small to moderate prostate volume. For detecting BPO, the model achieved an accuracy rate of 82%, without the need for invasive urodynamic study, thereby supporting the use of the two-stage model decision-making framework. We also constructed nomogram to broadly screening BOD by the combination of Qmax, voided volume, IPP, and PUA, a total score of ≥107 yielded the probability of 0.78 to identify BOD of 0.78. Subsequently, by combining PVR, TPV, and IPP, a total score of ≥39 yielded the probability of 0.35 to discriminate BPO. With two-stage model decision-making framework, male patients with LUTS can be screened for BPO and appropriately treated. The other BOD subtypes may need videourodynamic study to clarify the underlying pathophysiology and treated.

In previous studies, only one-third of men with LUTS were found to have BPO [2]. The other patients might have bladder dysfunction without BOO, such as HSB, DU, and DO. If these patients were treated with medication or surgery for BOO, the treatment outcome might be suboptimal in those without BOO [15]. In patients with BOD, BND, DV, and PRES are also common subtypes of LUTD, in addition to BPO [2,3,4]. Patients with BND can benefit from a transurethral incision of the bladder neck, without the need for total prostate tissue resection [16]. PRES or DV is frequently caused by central or peripheral urethral sphincter hyperactivity [17]. TURP might not effectively alleviate LUTS because the underlying cause for LUTS is not prostatic obstruction. On the contrary, patients might have LUTS that exacerbated after TURP, resulting in prolonged dysuria or urinary retention [18]. Therefore, a precise diagnosis and an appropriate treatment strategy for LUTD in men with LUTS are important.

VUDS is a gold-standard diagnostic tool for complicated LUTS or before surgical intervention in patients with a low prostatic volume or an undetermined cause for medical refractory LUTS [4]. However, because of invasiveness and irradiation exposure, VUDS has not been widely applied to investigate the precise LUTD in men with LUTS refractory to medical treatment. However, our previous research has shown that men with refractory LUTS have different LUTD including non-BOO, BPO, BND, DV, and PRES. Treatment based on an accurate diagnosis can prevent economic wasting, increase therapeutic effectiveness, and prevent unnecessary surgery and postoperative complications [17]. In light of these considerations, the International Consultation on Incontinence Research Society (ICI-RS) suggested that men with medically refractory LUTS or those planning to undergo surgery should undergo VUDS to ensure that the proceeding treatment is harmless and effective [19,20].

Based on the VUDS diagnosis, the study results revealed that only 28.3% (*n* = 87) of patients presented with prostate-related BOO (BPO), 39.1% (*n* = 120) with BND, 6.8% (*n* = 21) with PRES, and 7.8% (*n* = 24) with DV. Further, 17.9% (*n* = 55) of the patients were diagnosed with non-BOO (including DO, DU, hypersensitive bladder, and normal tracing). Patients who had acute or chronic urinary retention, extremely enlarged prostate, and neurogenic LUTD were excluded from the analysis. The percentage of each BOD in this study represents the real-world practice of men with medium-sized prostate and LUTS, in which a highly heterogeneous patient subgroups of BOD exist and require a clear differentiation of the pathophysiology for their LUTDs.

Further, this study revealed that the BPO model had the best overall performance, including an AUC of 0.85, accuracy of 0.82, sensitivity of 0.75, specificity of 0.86, PPV of 0.74, and NPV of 0.87, in the training dataset. With this model, patients with BPO can be identified and receive medical or surgical treatment. For the BOD model, the training dataset achieved an AUC of 0.71, accuracy of 0.73, sensitivity of 0.77, and specificity of 0.55. Based on these results, the BOD model had a better performance in identifying positive cases. However, it might have a higher false-positive rate. In the predictive models for BND, DV, or PRES, the PPV was not high enough to obtain an accurate diagnosis. In this regard, an invasive VUDS might be necessary in cases where the medical treatments are not effective in relieving LUTS according to the initial diagnosis of BOD.

According to the models of this study, nomograms were developed to identify BOD and BPO and to exclude PRES in male patients with LUTS. For the BOD model, as indicated by the red dashed line, a total score of >107 corresponded to a predicted probability of >0.78, thereby indicating a likely diagnosis of BOD. In this model, the Qmax, voided volume, IPP, and PUA were selected as indicators of BOD, including BND, BPO, DV, and PRES. A lower Qmax (e.g., 15 mL/s) has long been considered to have anatomical or functional BOO [6]. Patients with an enlarged IPP and a higher PUA have been reported to have BPO or BND, regardless of the TPV [16,17,18]. Although most men with BOO have a small voided volume, patients with mild to moderate BPO or functional BOO might have a normal or slightly reduced bladder capacity. Taken together, 78% of men with LUTS might have BOD.

Although some predictors in the models, such as IPP, PUA, and TZI, were not statistically significant, they were retained in the final models due to their clinical relevance and contribution to the overall model fit. Previous studies have indicated that these variables may reflect anatomical bladder outlet obstruction, which impairs normal urinary flow and serves as a potential characteristic of bladder outlet obstruction [16,17,18]. Therefore, even though these predictors were not statistically significant, their inclusion enhances the clinical interpretability of the nomogram.

For the BPO model, as indicated by the red dashed line, a total score of >39 corresponded to a predicted probability of >0.35, indicating a likely diagnosis of BPO. In men with BOD, the model can predict the presence of BPO using a higher PVR, larger TPV, and enlarged IPP. BPO is characterized by anatomical obstruction attributed to BPH. Therefore, an enlarged TPV is an important indicator of BPO [21,22]. In addition, a large PVR indicates incomplete bladder emptying caused by BOO, and a higher IPP indicates mechanical obstruction at the bladder neck [23]. With one or more of these indicators, men with BOD can be diagnosed with BPO. Medical therapy with an α-blocker, with a 5-α-reductase inhibitor, or surgical treatment with TURP are feasible therapies for BPO.

In addition to BPO, the diagnosis of BND, DV, and PRES using noninvasive urological examinations is challenging. BND and PRES are commonly encountered in young men with LUTS [2]. Among men aged <50 years who have voiding dysfunction, BND was reported in 54%, PRES in 24%, and low detrusor contractility in the remainder [24]. BND and PRES were more common in patients aged <70 years and those with a TPV of <40 mL [2]. If these patients with non-BPO received TURP, the treatment outcome might not be satisfactory. Urological complications such as persistent dysuria, bladder neck contracture, and exacerbated urinary incontinence might develop [25]. In this regard, a diagnosis ruling out BND, DV, or PRES is mandatory.

DV is commonly observed in elderly men with LUTS. Previous studies have shown that central nervous system disorders have early urological features. In one study of multiple sclerosis, urinary urgency (62%) was the most common urinary symptom, followed by frequency (50.4%), urge incontinence (44.7%), and nocturia (33%) [26]. Patients with early-stage Parkinson’s disease may have storage LUTS and mild motor symptoms. Patients with Parkinson’s disease usually report nocturia, urgency, and difficulty in voiding and present with DO on urodynamic examination [27]. In patients with dementia with Lewy bodies, LUTS is more likely to be an early feature of the disease [28]. LUTS in patients with DV include storage and voiding symptoms. TURP in these patients without anatomical BOO usually results in exacerbated storage LUTS, with improvement in voiding LUTS [29]. Therefore, it is important to identify patients with DV who will not benefit from bladder outlet surgery.

Using younger age, large voided volume, small PVR, and small TZI as indicators, the model can obtain a diagnosis of PRES if the total score is >139, which corresponds to a predicted probability of >0.23. Patients with PRES might not satisfactorily respond to α-1 blocker therapy. Thus, they could be mistakenly diagnosed as having clinical BPO and undergo TURP, resulting in unpredictable complications or exacerbated LUTS [17]. The risk of PRES is low. However, patients with these indicators for PRES should be cautiously investigated, and surgical intervention must be avoided to prevent undesired complications and failed treatment outcome [17,30]. For non-BPO patients, VUDS is still required if medical treatment is not effective in relieving LUTS. Among these patients, a high percentage of men had BND and BPO after VUDS and might need further treatment with TUI-BN or TURP to effectively relieve their LUTS [30].

Non-BOD LUTDs comprised stable bladder, HSB, DO, and DU. The common parameters included a higher Qmax, lower TPV, small IPP, and small PUA. Patients with these parameters can be excluded from having BOD by the first-stage model. In elderly men, DU is one of the common causes of nonobstructive LUTS [31]. In elderly patients with chronic medical diseases such as diabetes mellitus, chronic heart failure, and neurological diseases (such as cerebrovascular accident, Parkinson’s disease, and dementia), chronic urinary retention caused by DU is frequently encountered and difficult to manage [32]. Patients with DU usually have a diminished bladder fullness or urgency sensation, and they present with insufficient detrusor contractility, resulting in incomplete bladder emptying. Patients with DU typically void with abdominal straining, and an intermittent flow pattern is often observed. The bladder may exhibit normal or diminished sensitivity to first sensation or urge sensation [33]. Some patients with DU and BPO can also benefit from TURP [34]. However, those with absent bladder fullness sensation and detrusor contractility might not achieve the goal of spontaneous voiding without a catheter [31]. Therefore, an accurate diagnosis of male LUTD is important in identifying appropriate treatment strategies and, more importantly, in planning surgical intervention.

Although the BOD model showed relatively high sensitivity in the test set, its low specificity (0.47) and negative predictive value (0.35) indicate limited reliability in ruling out BOD. These metrics are too low to accurately identify patients truly free of BOD. Therefore, the Stage I (BOD) model should be considered to serve as an initial screening and supportive step before Stage II analyses (BPO, DV, and PRES), rather than a tool for excluding BOD. In clinical application, it functions as a preliminary classifier to identify patients at higher risk who require further evaluation.

The Stage II (BPO) model, although having limited sensitivity (0.58), demonstrated relatively higher specificity and negative predictive value, making it more suitable for identifying patients with a higher likelihood of BPO. In contrast, patients predicted to be at low risk of BPO should still undergo invasive diagnostic testing, such as VUDS, according to clinical risk stratification. Therefore, the model should be regarded as a tool for clinical risk stratification or supportive screening, rather than a replacement for invasive diagnostic modalities such as VUDS.

The BND, DV and PRES models exhibited overfitting in the test set, mainly due to the small sample sizes and class imbalance. Although the SMOTE technique was applied to partially alleviate this issue, it was insufficient to improve the generalizability of the models. Therefore, based on the current data, these two models do not yet have clinical applicability, and invasive VUDS remain the gold standard for diagnosing related conditions.

The current study had several limitations. First, this is a single center retrospective study. Second, because we collected case with full clinical parameters and VUDS results, the collection period is over a long period; however, all the investigation tools, techniques, and interpretation criteria did not change. Third, patients with TPV of >60 mL are likely to have BPO rather than the other BOD subtypes. Because patients with BOD subtypes other than BPO usually have small to moderate prostate volume, we excluded the patients with TPV >60 mL to limit the patients for differential diagnosis. That is also the reason why the specificity of diagnosis of BPO or BOD by this two-stage model is not as high as previously reported criteria for BOO. The generalizability of the data-driven nomograms based on the results of this study might have been misleading. Forth, the case number was small, particularly in men diagnosed with DV (*n* = 24) and PRES (*n* = 21). The small case number might have limited the PPV in the test sets using the model. Nevertheless, after the dual-model evaluation, only the non-BPO group required further VUDS examination, thereby effectively reducing the need for invasive diagnostic procedures for male LUTS.

This is the first study trial to use non-invasive parameters to construct a nomogram in identifying BOD and BPO. Although the results do not meet the initial expectation, the study further emphasize the importance of VUDS in the differential diagnosis of male LUTS with small to moderate prostate volume. Finally, a true external validation of this diagnostic model is lacking, further study to validate the accuracy of this model from a different institution is mandatory.

## 5. Conclusions

This is the first study demonstrating that non-invasive clinical parameters can be utilized to develop predictive models for the early identification and stratification of patients with BOD and those suspected of having BPO. Although the two models demonstrate different diagnostic performances, their sequential application can serve as a clinical risk stratification and preliminary screening mechanism that assists clinical decision-making and helps clinicians identify non-BPO patients who require further VUDS evaluation, thereby enhancing the efficiency of invasive VUDS in clinical practice and avoiding incorrect t treatment and unnecessary prostatic surgery.

## Figures and Tables

**Table 1:** Baseline characteristics of the patients and distribution of videourodynamic diagnoses in the training and test sets.

		Total	Training Set	Test Set	*p*-Value
		307 (100%)	215 (70%)	92 (30%)	
**VUDS Diagnosis, *n* (%)**					
Non-BOD		55 (17.9)	38 (17.7)	17 (18.5)	0.866
BOD (BND/BPO/DV/PRES)		252 (82.1)	177 (82.3)	75 (81.5)	
BND	Yes	120 (47.6)	84 (47.5)	36 (48.0)	0.937
	No	132 (52.4)	93 (52.5)	39 (52.0)	
BPO	Yes	87 (34.5)	61 (34.5)	26 (34.7)	0.975
	No	165 (65.5)	116 (65.5)	49 (65.3)	
DV	Yes	24 (9.5)	17 (9.6)	7 (9.3)	0.947
	No	228 (90.5)	160 (90.4)	68 (90.7)	
PRES	Yes	21 (8.3)	15 (8.5)	6 (8.0)	
	No	231 (91.7)	162 (91.5)	69 (92.0)	0.901
**Characteristics, Mean** **±** **SD**					
Age (years)		67.8 ± 9.7	67.8 ± 9.8	67.9 ± 9.5	0.927
Qmax (mL/s)		10.2 ± 6.5	10.6 ± 7.0	9.4 ± 5.1	0.105
VoL (mL)		196.8 ± 124.1	200.1 ± 124.7	189.2 ± 123.0	0.482
PVR (mL)		44.1 ± 83.9	48.7 ± 96.3	33.5 ± 41.2	0.146
TPV (mL)		38.1 ± 19.5	39.2 ± 20.9	35.4 ± 15.7	0.119
TZI		0.40 ± 0.16	0.40 ± 0.16	0.42 ± 0.14	0.393
IPP (cm)		0.3 ± 0.6	0.3 ± 0.6	0.3 ± 0.5	0.390
PUA (degree)		26.3 ± 18.7	25.9 ± 18.8	27.4 ± 18.3	0.517

BOD: bladder outlet dysfunction, BND: bladder neck dysfunction, BPO: benign prostatic obstruction, DV: dysfunctional voiding, PRES: poor relaxation of external sphincter, Qmax: maximum flow rate, VoL: voided volume, PVR: post-void residual, TPV: total prostate volume, TZI: transition zone index, IPP: intravesical prostatic protrusion, PUA: prostatic urethra angle.

**Table 2:** Comparison of noninvasive baseline clinical parameters among the BOD subtypes.

	Non-BOD (*n* = 55)	BND (*n* = 120)	BPO (*n* = 87)	DV (*n* = 24)	PRES (*n* = 21)	*p*-Value ^#^
Age (years)	66.0 ± 11.0	67.5 ± 9.2	70.4 ± 8.0	68.8 ± 10.3	62.9 ± 12.1	0.007
Qmax (mL/s)	13.0 ± 8.1	9.7 ± 6.1	8.8 ± 4.3	11.1 ± 7.9	11.4 ± 7.9	0.002
VoL (mL)	200.8 ± 121.3	209.8 ± 122.3	167.1 ± 110.4	185.8 ± 122.0	247.4 ± 172.5	0.039
PVR (mL)	44.0 ± 115.4	36.9 ± 44.0	63.2 ± 113.7	28.5 ± 29.4	24.5 ± 31.5	0.117
TPV (mL)	31.4 ± 13.8	32.0 ± 11.5	54.0 ± 24.2	30.9 ± 8.8	32.4 ± 19.4	<0.001
TZI	0.36 ± 0.15	0.37 ± 0.14	0.50 ± 0.13	0.37 ± 0.13	0.31 ± 0.18	<0.001
IPP (cm)	0.10 ± 0.26	0.16 ± 0.40	0.74 ± 0.76	0.10 ± 0.29	0.17 ± 0.66	<0.001
PUA (degree)	17.4 ± 17.2	25.9 ± 17.8	36.2 ± 15.5	16.5 ± 15.9	22.6 ± 23.2	<0.001

BOD: bladder outlet dysfunction, BND: bladder neck dysfunction, BPO: benign prostatic obstruction, DV: dysfunctional voiding, PRES: poor relaxation of external sphincter, Qmax: maximum flow rate, VoL: voided volume, PVR: post-void residual, TPV: total prostate volume, TZI: transition zone index, IPP: intravesical prostatic protrusion, PUA: prostatic urethra angle. ^#^: one-way ANOVA among five groups.

**Table 3:** Logistic regression models for predicting BOD subtypes.

	OR	95% CI	*p*-Value	Hosmer-Lemeshow *p*-Value
**Model 1: BOD (BND/BPO/DV/PRES)**				
Qmax (mL/s)	0.925	0.872–0.982	0.010	0.407
Voided volume (mL)	1.004	1.000–1.008	0.045	
IPP (cm)	2.356	0.807–6.873	0.117	
PUA (degree)	1.018	0.995–1.042	0.118	
**Model 2: BND**				
TPV (mL)	0.964	0.939–0.990	0.006	0.484
IPP (cm)	0.475	0.205–1.100	0.082	
**Model 3: BPO**				
PVR (mL)	1.006	0.999–1.014	0.081	0.788
TPV (mL)	1.064	1.033–1.096	<0.001	
IPP (cm)	2.254	0.960–5.289	0.062	
**Model 4: DV (after SMOTE, ratio 1:4)**				
Qmax (mL/s)	1.105	1.015–1.209	0.025	0.381
Voided volume (mL)	0.990	0.983–0.995	0.001	
TPV (mL)	0.941	0.897–0.978	0.006	
TZI	20.051	0.689–704.728	0.087	
PUA (degree)	0.958	0.930–0.984	0.002	
**Model 5: PRES (after SMOTE, ratio 1:4)**				
Age (years)	0.920	0.876–0.960	<0.001	0.394
Voided volume (mL)	1.003	1.000–1.006	0.041	
PVR (mL)	0.988	0.974–0.998	0.048	
TZI	0.032	0.001–0.620	0.002	

DV and PRES models were developed using SMOTE to address class imbalance (DV/non-DV = 40:160; PRES/non-PRES = 41:162). The reported ORs and AUCs are based on the resampled datasets. SMOTE: synthetic minority over-sampling technique. BOD: bladder outlet dysfunction, BND: bladder neck dysfunction, BPO: benign prostatic obstruction, DV: dysfunctional voiding, PRES: poor relaxation of external sphincter, Qmax: maximum flow rate, PVR: post-void residual, TPV: total prostate volume, TZI: transition zone index, IPP: intravesical prostatic protrusion, PUA: prostatic urethra angle, OR: odds ratio AIC: Akaike information criterion, CI: Confidence interval.

**Table 4:** Diagnostic performance of the prediction models for the BOD subtypes in the training, internal validation, and test sets.

	Stage I Model	Stage II Model			
	BOD	BND	BPO	DV	PRES
**Training set**					
AUC	0.71	0.70	0.85	0.84	0.83
Threshold of training set	0.78	0.40	0.35	0.17	0.23
Accuracy	0.73	0.68	0.82	0.72	0.81
Sensitivity	0.77	0.88	0.75	0.95	0.81
Specificity	0.55	0.49	0.86	0.66	0.81
PPV	0.89	0.61	0.74	0.41	0.52
NPV	0.34	0.82	0.87	0.98	0.94
AUC	0.70 ± 0.10	0.70 ± 0.07	0.84 ± 0.07	0.80 ± 0.06	0.81 ± 0.08
Accuracy	0.74 ± 0.06	0.66 ± 0.06	0.81 ± 0.06	0.68 ± 0.07	0.78 ± 0.06
**Test set**					
AUC	0.70	0.68	0.79	0.61	0.73
Accuracy	0.74	0.60	0.73	0.63	0.80
Sensitivity	0.80	0.83	0.58	0.71	0.33
Specificity	0.47	0.38	0.82	0.62	0.84
PPV	0.87	0.56	0.63	0.16	0.15
NPV	0.35	0.71	0.78	0.95	0.94

DV and PRES models were developed using SMOTE to address class imbalance (DV/non-DV = 40:160; PRES/non-PRES = 41:162). The reported ORs and AUCs are based on the resampled datasets. SMOTE: synthetic minority over-sampling technique. BOD: bladder outlet dysfunction, BND: bladder neck dysfunction, BPO: benign prostatic obstruction, DV: dysfunctional voiding, PRES: poor relaxation of external sphincter, PPV: positive predictive value, NPV: negative predictive value, AUC: area under curve

## Data Availability

The original contributions presented in this study are included in the article/Supplementary Materials. Further inquiries can be directed to the corresponding author.

## Supplementary Materials

The following supporting information can be downloaded at https://www.mdpi.com/article/10.3390/biomedicines13122894/s1; Figure S1: Receiver operating characteristic (ROC) curves of the multivariable logistic regression models. Table S1: Baseline Noninvasive Clinical Characteristics Stratified by the VUDS Diagnosis; Table S2. The diagnostic sensitivity and specificity of diagnosis of BPO using s single non-invasive clinical parameter.
