# Supplementary material for: Predicting Prostatic Obstruction and Bladder Outlet Dysfunction in Men with Lower Urinary Tract Symptoms and Small-to-Moderate Prostate Volume Using Noninvasive Diagnostic Tools
Source: Biomedicines. 2025 Nov 27;13(12):2894. doi: 10.3390/biomedicines13122894 (PMC12730589; doi:10.3390/biomedicines13122894)
Supplement: Supplementary file 1 [file biomedicines-13-02894-s001.zip › biomedicines-3905586-Supplemental materials.pdf]

Supplementary Materials

Table S1. Baseline Noninvasive Clinical Characteristics Stratified by the VUDS Diagnosis.

	**Non-BOD (n=55)**				**BOD (n=252)**				
	Stable (n=8)	HSB (n=6)	DO (n=28)	DU (n=13)	BND (n=120)	BPO (n=87)	DV (n=24)	PRES (n=21)	*p*-value
Age (years)	65.4±7.0	60.4±12.3	65.4±10.7	70.2±12.6	67.5±9.2	70.4±8.0	68.8±10.3	62.9±12.1	0.009
Qmax (ml/s)	11.0±3.7	18.0±7.8	14.2±9.1	9.4±6.6	9.7±6.1	8.8±4.3	11.1±7.9	11.4±7.9	<0.001
VoL (ml)	280±169.9	260±123.7	178.0±101.0	173.7±109.9	209.8±122.3	167.1±110.4	185.8±122.0	247.4±172.5	0.021
PVR (ml)	50.5±58.7	14.3±11.3	24.3±31.8	96.3±226.4	36.9±44.0	63.2±113.7	28.5±29.4	24.5±31.5	0.037
TPV (ml)	36.0±14.0	27.2±18.4	32.9±15.0	27.3±7.6	32.0±11.5	54.0±24.2	30.9±8.8	32.4±19.4	<0.001
TZI	0.35±0.11	0.20±0.13	0.40±0.12	0.38±0.21	0.37±0.14	0.50±0.13	0.37±0.13	0.31±0.18	<0.001
IPP (cm)	0.13±0.23	0.08±0.20	0.08±0.22	0.15±0.38	0.16±0.40	0.74±0.76	0.10±0.29	0.17±0.66	<0.001
PUA (degree)	23.8±17.5	20.0±20.7	13.8±16.7	20.0±16.7	25.9±17.8	36.2±15.5	16.5±15.9	22.6±23.2	<0.001

BOD: bladder outlet dysfunction, BND: bladder neck dysfunction, BPO: benign prostatic obstruction, DV: dysfunctional voiding, PRES: poor relaxation of external sphincter, Qmax: maximum flow rate, VoL: voided volume, PVR: post-void residual, TPV: total prostate volume, TZI: transition zone index, IPP: intravesical prostatic protrusion, PUA: prostatic urethra angle.

Table S2. The diagnostic sensitivity and specificity of diagnosis of BPO using s single non-invasive clinical parameter.

Clinical parameter N (interclass%)		Non-BOD (n=55)	BND (n=120)	BPO (n=87)	DV (n=24)	PRES (n=21)	Specificity of BPO
**TPV (ml)**	≥ 40	11 (20.0)	26 (21.7)	58 (66.7)	3 (12.5)	4 (19.0)	56.9%
	< 40	44 (80.0)	94 (78.3)	29 (33.3)	21 (87.5)	17 (81.0)	
**Qmax (ml/s)**	< 10	23 (41.8)	72 (60.0)	49 (56.3)	12 (50.0)	11 (52.4)	29.3%
	≥ 10	32 (58.2)	48 (40.0)	38 (43.7)	12 (50.0)	10 (47.6)	
**PVR (ml)**	≥100	5 (9.1)	11 (9.2)	15 (17.2)	1 (4.2)	1 (4.8)	45.5%
	<10	50 (90.9)	109(90.8)	72 (82.8)	23 (95.8)	20 (95.2)	
**PUA(degree)**	≥ 30	18 (32.7)	70 (58.3)	74 (85.1)	7 (29.2)	11 (52.4)	41.1%
	< 30	37 (67.3)	50 (41.7)	13 (14.9)	17 (70.8)	10 (47.6)	
**IPP (cm)**	≥ 0.5	7 (12.7)	22 (18.3)	54 (62.1)	3 (12.5)	2 (9.5)	61.4%
	< 0.5	48 (87.3)	98 (81.7)	33 (37.9)	21 (87.5)	19 (90.5)	
**Pdet (cmH2O)**	≥50	3 (5.5)	53 (44.2)	71 (81.6)	7 (29.2)	0	53.0%
	<50	52 (94.5)	67 (55.8)	16 (18.4)	17 (70.8)	21 (100)	
**BOOI**	≥ 40	1 (1.8)	30 (25.0)	48 (55.2)	3 (12.5)	0	58.5%
	< 40	54 (98.2)	90 (75.0)	39 (44.8)	21 (87.5)	21 (100)	

BOD: bladder outlet dysfunction, BND: bladder neck dysfunction, BPO: benign prostatic obstruction, DV: dysfunctional voiding, PRES: poor relaxation of external sphincter, Qmax: maximum flow rate, PVR: post-void residual, TPV: total prostate volume, IPP: intravesical prostatic protrusion, PUA: prostatic urethra angle, Pdet: detrusor pressure, BOOI: bladder outlet obstruction index

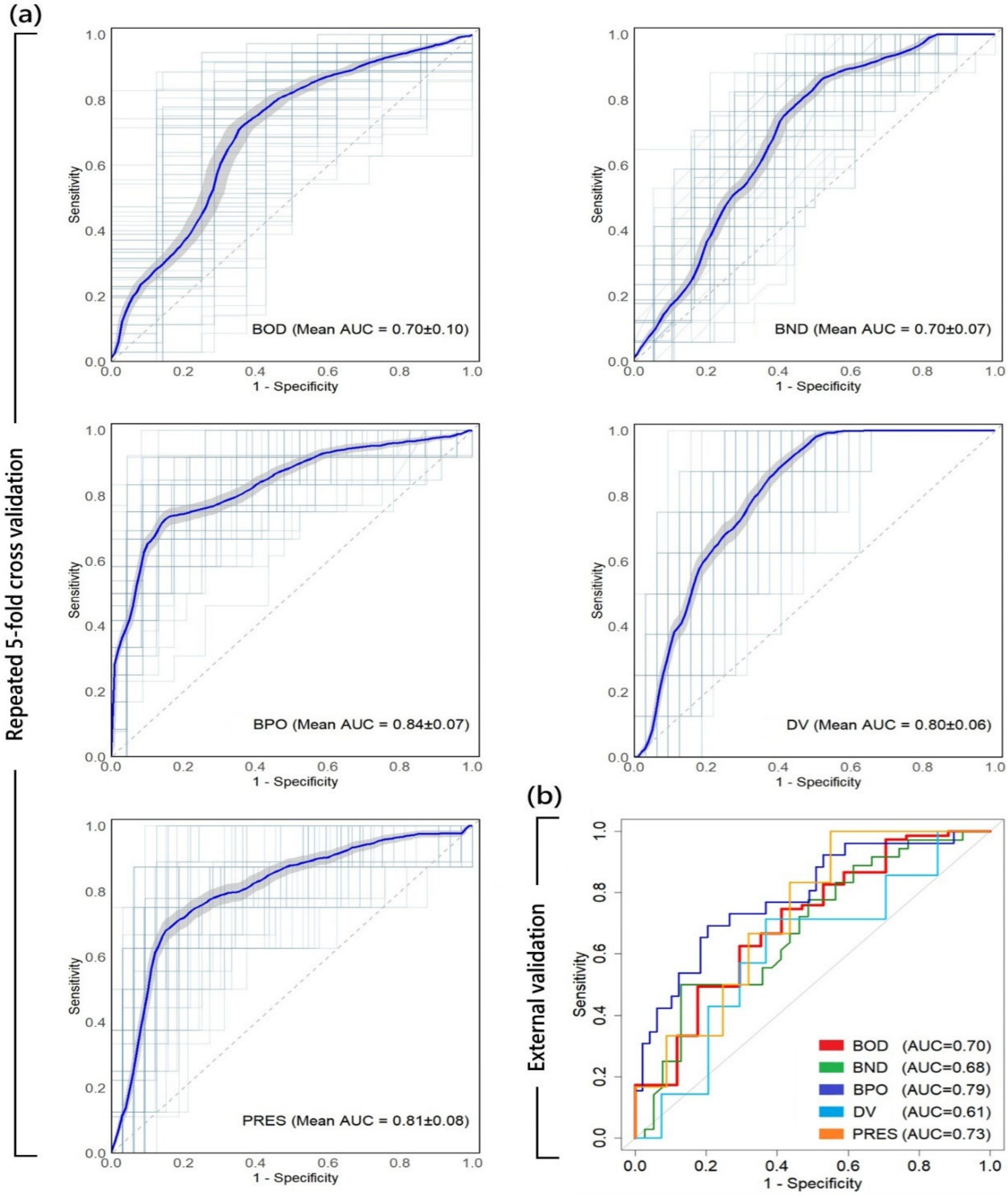

Figure S1. Receiver operating characteristic (ROC) curves of the multivariable logistic regression models. (a) ROC curves for five classification models—BOD, BND, BPO, DV, and PRES—based on repeated 5-fold cross-validation in the training set. The blue line represents the mean ROC across folds, and the shaded area indicates the standard deviation (SD). The AUC values (mean ± SD) are shown at the bottom of each panel. (b) ROC curves generated from the independent test set for external validation of five classification models. BOD: bladder outlet dysfunction; BND: bladder neck dysfunction; BPO: benign prostatic obstruction; DV: dysfunctional voiding; PRES: poor relaxation of external sphincter.
